# Temporal Links in Daily Activity Patterns between Coral Reef Predators and Their Prey

**DOI:** 10.1371/journal.pone.0111723

**Published:** 2014-10-29

**Authors:** Yoland J. Bosiger, Mark I. McCormick

**Affiliations:** ARC Centre of Excellence for Coral Reef Studies, and School of Marine and Tropical Biology, James Cook University, Townsville, Queensland, Australia; University of Ferrara, Italy

## Abstract

Few studies have documented the activity patterns of both predators and their common prey over 24 h diel cycles. This study documents the temporal periodicity of two common resident predators of juvenile reef fishes, *Cephalopholis cyanostigma* (rockcod) and *Pseudochromis fuscus* (dottyback) and compares these to the activity and foraging pattern of a common prey species, juvenile *Pomacentrus moluccensis* (lemon damselfish). Detailed observations of activity in the field and using 24 h infrared video in the laboratory revealed that the two predators had very different activity patterns. *C. cyanostigma* was active over the whole 24 h period, with a peak in feeding strikes at dusk and increased activity at both dawn and dusk, while *P. fuscus* was not active at night and had its highest strike rates at midday. The activity and foraging pattern of *P. moluccensis* directly opposes that of *C. cyanostigma* with individuals reducing strike rate and intraspecific aggression at both dawn and dusk, and reducing distance from shelter and boldness at dusk only. Juveniles examined were just outside the size-selection window of *P. fuscus*. We suggest that the relatively predictable diel behaviour of coral reef predators results from physiological factors such as visual sensory abilities, circadian rhythmicity, variation in hunting profitability, and predation risk at different times of the day. Our study suggests that the diel periodicity of *P. moluccensis* behaviour may represent a response to increased predation risk at times when both the ability to efficiently capture food and visually detect predators is reduced.

## Introduction

Determining the patterns of activity of predators and their prey is central to understanding predator-prey dynamics [1]–[3]. The threat of predation exerts a strong selective force on prey, influencing everything from morphology and life history to behaviour (for reviews see [4], [5]). Predation risk may vary greatly between habitats and through time [1]. On a temporal scale, predator activity patterns may vary across lunar cycles [6], seasons [7], or within days [8]. However, the degree to which predation risk varies predictably over a daily temporal scale, is not well understood in most ecological systems [9].

The simple diel cycle of the rising and setting of the sun imposes an overriding set of constraints on the behaviour and activity of most animals [10]. Hence, animals often have activity schedules that are synchronized to relatively predictable diel cycles [11]–[13]. Activity patterns may vary substantially among different species, and within a 24 h day animals are commonly described as being diurnal, nocturnal, or crepuscular [11], [14], [15]. Such differences in predominant diel activity may relate to an animal's physiology, the presence of competitors, predation risk, and prey availability [12], [16].

Foraging periodicity can be linked to the profitability of feeding and optimal foraging theory proposes that predators should forage in patches when prey density is high [17]. However, if we accept that both predators and prey are capable of altering their behaviour in response to one another [18], mathematical game theory can be used to predict the strategies that each party will employ to best optimize catch success [19]. For example, the best predatory tactic might be to forage on an unpredictable schedule in order to decrease the possibility that diel activity patterns would be anticipated by prey [20]. Despite the apparent benefit of such foraging tactics, physiological tolerance and developmental factors, as well as resource partitioning through evolutionary time, may prohibit predators from displaying such unpredictable strategies [9], [16]. Indeed, visual capabilities [21], [22], temperature regulatory mechanisms [23] and/or genetically predisposed hunting strategies [24] may force predators to be relatively predictable in their diel activity patterns.

On coral reefs, extensive anecdotal evidence has accumulated on the activity patterns in piscivorous fishes [10], [25]–[27]. While there is some evidence of plasticity in the activity patterns of freshwater and diadromous species [28], [29], coral reef fishes are usually assumed to be fixed in their daily activity patterns [14], [30], [31]. In particular, the visual sensory system of many coral reef fishes may effectively force individuals to be active at either high or low light levels [10], [25], [32]. On coral reefs, families thought to be diurnally active include the wrasses (Labridae) and trevallies (Carangidae), while nocturnal fishes include grunts (Haemulidae) and squirrelfishes (Holocentridae) [33]. In contrast, crepuscular fishes comprise species which remain active during the transition between day and night, and those that forage primarily at dawn and dusk, such as groupers (Serranidae) and snappers (Lutjanidae) [34]. Where predation pressure is relatively predictable there may be potential for these patterns to influence the activity patterns of prey.

Predictable variation in predation risk over a daily scale has been implicated to mediate patterns of activity [35] and reproduction in prey animals [36] in coral reef environments. For example, increased diurnal predation pressure in reef environments is thought to drive reef-based populations of the rabbitfish, *Siganus lineatus*, to forage only during nocturnal hours. In contrast, shoreline populations of *S. lineatus*, which are thought to experience less diurnal predation risk, forage during the day and remain stationary at night [35]. Furthermore, many coral reef predators are thought to have visual capabilities suited to the low-light of crepuscular hours [21], and this twilight risk is thought to influence the dawn and dusk sheltering time of diurnal prey fish in coral reefs around the world [37], [38].

Small-bodied resident predators have been widely acknowledged for their importance in coral reef environments [37], [39], [40]. These mesopredators are responsible for a substantial amount of juvenile mortality at and shortly after settlement, and have been acknowledged to exert a significant influence over the community composition of prey fishes [37], [41]. Spatial and temporal variability in risk exerted by these predators at settlement and after settlement may therefore strongly influence the behaviour of prey fishes [37], [42]. While small-scale spatial variation in resident predators is widely recognised on coral reefs [43]–[45], few studies have attempted to quantify their temporal variability in activity patterns, and particularly those over a diel scale (but see [46]–[48]).

The present study investigated the diel foraging and activity patterns of two small-bodied, coral reef resident predators: *Cephalopholis cyanostigma* (Serranidae: rockcod) and *Pseudochromis fuscus* (Pseudochromidae: dottyback). Predator activity patterns were then compared to the temporal activity and behavioural patterns of juveniles of a common prey species, the lemon damselfish *Pomacentrus moluccensis* (Pomacentridae). Activity was documented through a combination of direct focal observations in the field and controlled laboratory studies. Purpose-built tanks that constantly provided prey cues to the two predators allowed activity to be quantified over a 24 h period under standardised conditions and permitted meaningful comparison of activity patterns to be made between species. At the time of the study, the juvenile prey were slightly larger than the size window targeted by the smaller predatory *P. fuscus*, but within the size-selection window of *C. cyanostigma* [49]. Therefore, our prediction was that activity patterns of *P. moluccensis* would be more likely to oppose the activity patterns of the relevant common predator *C. cyanostigma*, rather than *P. fuscus*.

## Materials and Methods

### Study species and site

The study was conducted at Lizard Island (14°40′S, 145°28′E), in the northern Great Barrier Reef (GBR), Australia during the summer fish recruitment season. The two predator species investigated were *C. cyanostigma* and *P. fuscus*. Both are widely distributed throughout the Indo-Pacific and are considered important predators on juvenile coral reef fishes [43]. Diel activity information for *P. fuscus* was collected using the same techniques as that for *C. cyanostigma*, but has been already included in a more detailed study of the species' foraging ecology [39]. Data for *P. fuscus* is included in the present study as a direct comparison of the activity patterns of two important predators of juveniles to illustrate the potentially different patterns of risk that prey must cope with during their juvenile life. Field data for *C. cyanostigma* were collected one year after that for *P. fuscus*, when temporal constraints on sampling were more relaxed (due to the experience of personnel). The slight methodological differences used for *P. fuscus* (compared to that for *C. cyanostigma*) are briefly described to aid comparison. Areas chosen for behavioural studies were those sites where the predator species were common. Weather conditions meant that sampling occurred in Lizard Island lagoon and lagoonal back-reef. These also happened to be sites where juvenile *Pomacentrus moluccensis* (lemon damselfish) (Bleeker 1968) were abundant.

*Pomacentrus moluccensis* are one of the most abundant planktivorous damselfish around Lizard Island, are strongly associated with live branching corals [43] and are common across the Indo-Pacific. The damselfish feeds primarily on algae and zooplankton [50] and are preyed upon by both *P. fuscus* and *C. cyanostigma* [43], [51]. *P. moluccensis* are also highly site attached making them ideal for behavioural observations [51], [52].

### Ethic statement

This research was carried out in accordance with James Cook University ethics guidelines under ethics approval A1067 and conducted in accordance with the Queensland Department of Primary Industries collection permit (103256) and a Great Barrier Reef Marine Park Authority research permit (G09/29995.1).

### Behaviour of predators in the field

#### Focal observations

To determine the diel periodicity of activity patterns of *C. cyanostigma*, 70 replicate focal observations were made on individuals (mean TL = 212 mm, ranging 120 to 320 mm TL) in the field on SCUBA for periods ranging from 18–30 min (29.4±0.31, mean ± SE, total 2057 min) during February to April 2011. No individual was observed more than once during a particular time period to maintain the independence of replicates. Observations were undertaken at three distinct time periods: dawn (06:00–08:00 h, n = 26), midday (11:30–13:30 h, n = 21) and dusk (17:00–19:00 h, n = 23). Upon entering the study site, the first *C. cyanostigma* located was observed at a minimum distance of 2 m. This distance caused no apparent stress to the fish and was similar to that used by Sweatman (1984) [46] and Feeney et al. (2012) [39] for other reef predators. The total length (±10 mm) of each fish was estimated by noting the position of the tip of the snout and the end of the caudal fin relative to points on the substratum [46].

The number of predation attempts by focal individuals during the observational period was quantified by recording the number of feeding strikes. Where possible, strikes were categorised as being directed towards either fish or invertebrate prey items as previous studies have identified fish, small crustaceans, molluscs and eggs in the gut contents of *C. cyanostigma* [51]. Whether strikes were successful or unsuccessful could not be reliably distinguished.

The activity of individual *C. cyanostigma* was determined by continuously recording the time spent stationary, hiding, or swimming throughout the focal observation. *C. cyanostigma* was defined as hiding when >0.5 of its body length was concealed within the reef matrix, stationary when ≥0.5 of its body was outside the reef matrix and it maintained its position either in the water column or on the reef substratum, and swimming when actively moving position. The distance moved was also estimated each time a fish was seen swimming in order to accurately assess the total distance moved during an observation period. The distance moved was defined as the distance between the position at the midpoint of the fish's body (start) and the mid-point at the finish of each move [46].

Aggressive interactions were assessed by recording the number of fin displays, chases, bites and avoidance episodes in response to conspecifics and heterospecifics. An established aggression index was used, calculated by adding the number of displays to the product of three times the number of aggressive chases/bites and then subtracting the number of avoidance events [53]. Chases and bites appeared to influence the spatial distribution of recipients much more than displays, and therefore the weighting factor of three used by McCormick (2009) [53] was deemed suitable.

Behavioural observations were conducted on *P. fuscus* in the shallow reef (2–4 m) surrounding Lizard Island in December 2009. During this period 20 individual *P. fuscus* were observed for periods ranging from 55 to 75 min (mean ± SE = 61.65±1.06 min, total 1,233 min). Due to logistical constraints at the time, observations were performed between 08:00 and 17:00 h, effectively excluding dawn and dusk periods for the field observations of this species. Focal animal observations were conducted as for *C. cyanostigma* with all strikes directed at fish or substratum (probably invertebrates) recorded separately. Because of diving logistics at the time of the study, observations were divided morning (08:00–11:00 h; n = 6), midday (11:00–14:00 h; n = 6) and afternoon (14:00–17:00 h; n = 8). Note that these are broader time windows than those used for the *C. cyanostigma* field study.

#### Patterns of abundance of C. cyanostigma

The diel abundance of visible *C. cyanostigma* was recorded on snorkel using visual census. Due to logistical constraints this was carried out 2 months after the behavioural observations of *C. cyanostigma* were conducted at the same location. Changes in day length necessitated the slight modification of time periods to the following: dawn (07:00–08:00 h), midday (11:30–13:00 h), and dusk (16:00–18:00 h). Sixteen 20×2 m visual strip-transects were conducted during each time period, run parallel to the outer edge of the reef. *C. cyanostigma* were counted by the observer whilst laying the tape to minimise diver disturbance. When conducting multiple transects, independence of replicates was maintained by ensuring a random gap of at least 10 m separated transects. A 2 m long plastic rod was used to calibrate the single observer's definition of transect width in the field prior to census.

### Behaviour of predators in the laboratory

#### Collection and maintenance

Both *C. cyanostigma* (184.5±3.8 mm TL, mean ± SD) and *P. fuscus* (69.8±4.6 mm TL, mean ± SD) were collected from around Lizard Island: *C. cyanostigma* was captured using baited hook and line on snorkel, and *P. fuscus* were collected on SCUBA using hand nets and a solution of the anesthetic clove oil (10%), alcohol and seawater. Fish were then held in 16 l aquaria with flow-through aerated seawater and acclimated for a minimum of 24 h. Both *C. cyanostigma* and *P. fuscus* were fed thawed squid once daily and feeding times were randomised to ensure that predators did not learn to be more active at a particular time of the day.

#### Observation tanks

**Figure 1:**
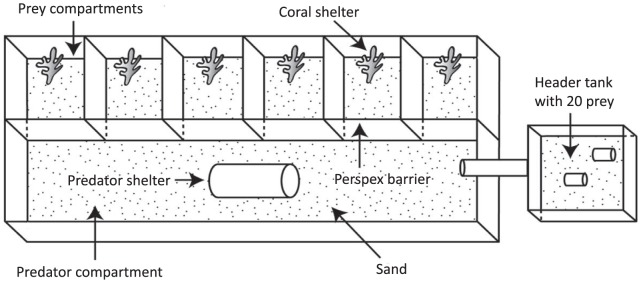
Schematic illustration of a predator-prey tank comprising one predator compartment and six prey compartments. Shelter consists of a plastic tube for each predator and a *Pocillopora* coral for each prey. Predators receive olfactory cues from a header tank containing 20 prey individuals and visual cues through a Perspex barrier separating predators from prey. For simplicity only a tank for *Pseudochromis fuscus* is shown in the figure.

Observations of the temporal periodicity of predators were conducted in predator-prey tanks (Fig. 1). A single predator-prey tank consisted of a single large compartment (predator compartment) and adjacent small compartments (prey compartments) (Fig. 1) The large compartment was separated from the smaller compartments by Perspex so as to allow predators and prey to observe each other without any physical interaction. Prey used were juvenile *P. moluccensis* collected with hand-nets from the reef.

Two different sized predator-prey tanks were used to take into account the difference in average size of *P. fuscus* and *C. cyanostigma*. Individual *C. cyanostigma* were transferred into a 60 l (34×73×24.5 cm) predator-prey tank with a 45 l (25×73×24.5 cm) predator compartment and four, 4 l (9×18.2×24.5 cm) prey compartments. *P. fuscus* were transferred into smaller 23 l (19.5×65×17.5 cm) predator-prey tanks with an 11 l (9.5×65×17.5 cm) predator compartment and six, 2 l (10.8×10×17.7 cm) prey compartments (Fig. 1). Each predator-prey tank had a 2 cm layer of sand spread across the bottom of both predator and prey compartments. Predator compartments had one plastic tube placed in the center of the compartment to provide shelter (Fig. 1). Similarly, a single dead *Pocillopora* coral (approximately 3×3×4 cm) was placed at the back of each prey compartment to create a shelter for prey. Predator-prey tanks were situated outside to ensure that all potentially necessary temporal cues (e.g. sun position, temperature) were available to the test fish. To ensure that predators received important prey odour cues in addition to visual cues continuously, a 32 l header tank (43×32×30 cm) containing 20 prey fish delivered seawater into the predator-prey tank (Fig. 1).

#### Laboratory protocol

At the commencement of the study, each prey compartment was stocked with two individual prey fish. An individual predator was transferred into the predator compartment and acclimated for 24 h in an attempt to reduce the confounding influence of stress induced through capture, handling and a new environment on the temporal patterns in their behaviour. Following acclimation, predators were filmed for 24 h using a bullet camera (Solex Model S 9139) positioned 80 cm above the middle of the predator-prey tank so it recorded the entire tank. The camera was infrared capable with infrared emitting diodes surrounding the lens making it extremely low-light sensitive and capable of recording video at night. Predators were fed just prior to being released into predator-prey tanks but were not fed for the duration of the experiment (48 h) to avoid increases in activity due to feeding. As predators were fed just prior to each trial, individual activity levels and foraging could simply increase as a result of rising hunger over the 24 h period. To reduce this potentially confounding factor, predators were released into predator-prey tanks at one of two times of the day: 10:30 h or 16:00 h. Predators were thus at their maximum hunger level at one of two different times, and statistical tests were used to determine whether a difference in diel activity pattern existed between predators started (and hence fed) at the two different times. Prey fish in prey compartments and in the header tank were fed *Artemia* nauplii ad libitum twice daily and care was taken to randomise feeding times to ensure that increased predator activity was not due to the feeding of prey. Overall a total of 10 replicate *C. cyanostigma* and 10 replicate *P. fuscus* individuals were video recorded for 24 h each.

#### Behavioural assay

A pilot study found that a sample of 10 min per hour of video gave an accurate measure of hourly activity and foraging, while 20 min of video per hour was required for *C. cyanostigma*. Periods analysed were the first 10 and 20 min of each hour. Overall, 24 subsamples of 20 min duration were watched per 24 h replicate for *C. cyanostigma* (80 h total), and 24 subsamples of 10 min duration were watched per replicate for *P. fuscus* (40 h total).

Foraging rate and activity level were recorded for both *C. cyanostigma* and *P. fuscus* for all hours in the 24 h period. Foraging rate was recorded as the total number of strikes at prey compartments per time period. A predatory strike was distinguished from normal motion by speed (movement greater than 2.5 body lengths per second). Usually a strike involved the predators touching a prey compartment with its nose. However, to avoid any possible effect of habituation throughout the 24 h, a strike was also counted in the absence of a touch provided the predator made a fast movement (similar to that observed in the field) and its head came within 2 cm of the prey compartment. The activity level of predators was determined by recording the proportion of time spent hiding, swimming and stationary. As *P. fuscus* rarely remains stationary, only time spent hiding and swimming were recorded for this species. Definitions of hiding, swimming and stationary were identical to those used during the field observations.

The proportion of time spent swimming per hour was determined by dividing the time spent swimming by the total time in each subsampling period. Average activity levels were relatively consistent among individuals over the 24 h period and therefore activity levels were left as raw hourly values to demonstrate the consistency among individuals. However, there was high inter-individual variation in the total number of strikes made over 24 h. To avoid this from obscuring potentially consistent diel foraging patterns, hourly strike rate was divided by the total number of strikes (measured within the hourly subsets) that individual made over the 24 h. Behaviour of the prey in the tanks adjacent to the predator tank was not quantified because the videos did not have sufficient resolution to obtain accurate information on these much smaller fish.

### Behaviour of prey in the field

The behaviour of individual *P. moluccensis* (25.02±4.48 mm TL, mean ± SD) was documented in conjunction with observations of *C. cyanostigma* by a single observer at dawn, midday and dusk (n = 32, dawn; 29, midday; 26, dusk). Behaviour of each replicate individual was assessed over a 3 min period, which is sufficient to accurately determine foraging rates [54] and other behaviours of interest for this species [55].

Each focal *P. moluccensis* was located in a separate haphazardly chosen natural coral patch. As individuals within a patch may vary in personality, care was taken to ensure that the most bold individual within the coral head was chosen (defined in next paragraph), therefore minimizing the potential that different personalities might confound results between times periods. Size of social groups may also influence the intensity of antipredator behaviour and therefore individuals were chosen only from social groups containing at least 4 prey. Scuba divers were positioned at least 1.5 m away from the coral patch to avoid disturbing fish.

*P. moluccensis* behaviour was determined using a well-established behavioral protocol [53], [54]. Eight aspects of activity and behaviour were assessed: a) strike rate b) total distance moved; c) distance ventured from coral patch (categorized as % of time spent 0–2, 2–5, and 5–10 cm from shelter); d) maximum horizontal distance ventured from coral patch; e) number of fin displays in response to a conspecific; f) the number of chases or bites in response to a conspecific; g) number of avoidance episodes in response to a conspecific, and h) boldness, which was recorded on a scale from 0 to 3 with 0.5 increments, where: 0 is sheltering and seldom emerging; 1 is sheltering and taking more than 5 sec to re-emerge, and weakly or tentatively striking at food; 2 is sheltering when scared but quickly emerging, and purposefully striking at food; and 3 is not hiding when scared, exploring around the coral patch, and striking aggressively at food.

Two further variables were created to summarise these behavioural measures [50]. Relative horizontal distance ventured from coral patch was calculated from the sum of the proportions of time spent in each of the distance categories multiplied by the distance that each category represented [54]. Secondly, an aggression index identical to that used for *C. cyanostigma* was employed for *P. moluccensis* [52].

### Statistical analysis

#### Behaviour of predators in the field

To test whether the behaviour of *C. cyanostigma* varied among the three focal periods of the day (morning, midday and dusk) a 1-factor MANOVA was used. Included in the MANOVA were four response variables: feeding strikes on fish (per min), the proportion of time spent swimming, total distance moved (per min), and aggression index. Residual analysis on raw data revealed that the data violated the assumptions of normality and homogeneity of variance for the aggression index and a log_10_(x+1) transformation was applied. ANOVAs and Tukey's HSD post-hoc comparisons were then conducted on each response variable to determine the nature of the differences found by MANOVA. All values given in the text and figures are the arithmetic means ±1 standard error (SE) of untransformed data.

To determine whether the number of predators visible in transects varied across the three time periods, a 1-factor ANOVA was conducted. A fourth-root transformation significantly improved the assumptions of normality and homogeneity of variance. Examination of residual plots indicated that there were no outliers or influential points in the dataset.

#### Behaviour of predators in the laboratory

To compare the behaviour of *C. cyanostigma* and *P. fuscus* in the predator-prey tanks a repeated measures ANOVA (RMANOVA) was conducted for each behavioural measure (proportion of strikes and proportion of time spent swimming). For each analysis, the assumption of sphericity of the variance–covariance matrix was tested using Mauchley's test [56]. This assumption was satisfied for proportion of strikes per time period and therefore split-plot RMANOVA (a univariate analog to the multivariate RMANOVA) was used to investigate interactions between species and time of day for this dependent variable. Significant differences among means were then explored using Tukey's HSD means comparison test. Mauchley's test revealed that the assumption of sphericity was violated for the proportion of time spent swimming. Therefore a multivariate RMANOVA approach (which does not assume sphericity of the variance–covariance matrix) was used, with time as the ‘within subjects’ factor and species as the ‘between subjects’ factor [56]. Tukey's post-hoc comparisons following multivariate RMANOVA are not advisable when the assumption of sphericity is violated [56] and thus examination of means and standard errors was used to draw tentative conclusion regarding where differences lay.

To ensure that there were enough degrees of freedom to use the multivariate RMANOVA approach [56], the within subject factor (Time) was averaged into eight 3 h time categories: night 1 (02:00–04:59 h), dawn (05:00–07:59 h), morning (08:00–10:59 h), midday (11:00–13:59 h), afternoon (14:00–16:59 h), dusk (17:00–19:59 h), night 2 (18:00–22:59 h), and midnight (23:00–01:59 h). The three-hour values within each time category were averaged to give the value for that category (except for the first time category after the video was started which only contained 2 h). Assumptions of normality and homogeneity of variance were explored using residual analysis and an arcsin square-root transformation was applied to improve normality and homogeneity of variances. For the proportion of strikes, deviations from homogeneity of variance were still seen for the nighttime periods (18:00–04:59 h) and therefore the univariate RMANOVA was conducted on the daytime periods (05:00–19:59 h) only.

Finally, to ensure that hunger did not influence the behaviour of predators during the study, a single multivariate RMANOVA was conducted on each of the two dependent variables (proportion of strikes and proportion of time spent swimming) with Time as the ‘within subjects’ factor and Time-started as the ‘between subjects’ factor on daytime periods (05:00–19:59 h). No violations of assumptions were observed in the residual analysis.

#### Behaviour of prey in the field

Prey behaviour at dawn, midday and dusk was analysed using a 1-factor MANOVA. Individual dependent variables (behaviours) where then investigated by exploring univariate ANOVA results followed by Tukey's HSD post hoc comparisons. Residual analysis revealed that the data slightly violated assumptions of normality and homogeneity of variance, and a cube-root transformation was applied to total distance moved and a log_10_ transformation was applied to maximum horizontal distance ventured.

The potentially confounding effects of fish size and water temperature on the behaviour of predators and prey in the field was examined using one-factor ANCOVAs. However, neither variable was found to account for a significant amount of variability in behaviour for any focal species. Temperature data was sourced from the Integrated Marine Observing System (IMOS).

Data for the figures can be found in the Tables S1–S4 in File S1.

## Results

### Behaviour of predators in the field

#### Focal observations

In a total of 2057 min of underwater focal observations, *C. cyanostigma* stuck at prey 117 times. Overall 63 strikes where directed at juvenile fish, 37 where directed at invertebrates and 17 were aimed at unidentifiable prey sheltering within coral. During the 30 min observations periods they travelled an average of 35.1 m, ranging from 1 m to 132 m. In contrast, in 1230 min of observation *P. fuscus* struck at 912 prey with 216 of these directed at juvenile fishes, the rest being directed to the substratum (and are assumed to be invertebrates).

**Figure 2:**
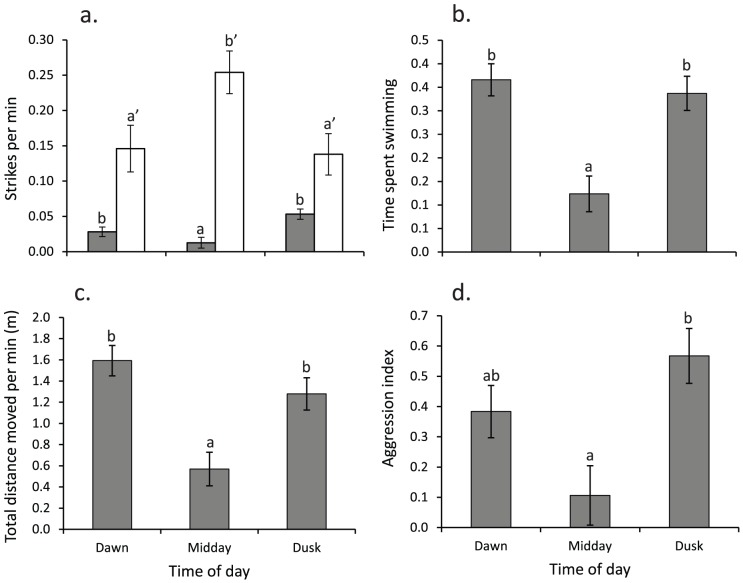
Diel variation in behaviour of *Cephalopholis cyanostigma* and *Pseudochromis fuscus* (means ± SE) for three times of the day: dawn, midday, and dusk. a) Total strikes per minute at fish for *C. cyanostigma* (dark grey bars) and *P. fuscus* (white bars), b) proportion of time spent swimming (as opposed to hiding and stationary) by *C. cyanostigma*, c) total distance (m) moved per minute by *C. cyanostigma*, and d) aggression index (+ aggression, − avoidance) for *C. cyanostigma*. Letters above bars represent Tukey's HSD homogenous subsets. N = 26, 21, 23 for dawn, midday and dusk respectively for *C. cyanostigma*, while N = 6, 6, and 8 for *P. fuscus*. Note that the definition of the time intervals for the two species differ slightly (see text for details). Data for *P. fuscus* in Fig. 2a from Feeney et al. (2012).

The overall behaviour of *C. cyanostigma* differed among dawn, midday, and dusk observation periods (MANOVA, Pillai's trace_10, 106_ = 0.624, p<0.0001). Separate ANOVA's conducted on each dependent variable revealed that strike rate on fishes (F_2, 67_ = 7.588, p = 0.001), the proportion of time spent swimming (F_2, 67_ = 12.933, p<0.0001) and total distance moved (F_2, 67_ = 11.606, p<0.0001) differed with time of day, while strike rate on invertebrates did not significantly change (F_2, 67_ = 2.600, p = 0.082). Post-hoc Tukey's HSD tests showed that strike rate on fishes was significantly (p<0.05) higher at dusk relative to other daytime periods, and activity (swimming and distance moved) was increased at dawn and dusk relative to midday (Fig. 2a, b, c). Levels of aggression changed with time of day (log_10_ transformed; F_2, 56_ = 6.00, p = 0.004), with more aggression shown at dusk than midday and an intermediate level of aggression at dawn (Fig. 2d). Strike rates of *P. fuscus* on fishes also differed with time of day (F_2, 17_ = 4.07, p = 0.04), with significantly more strikes occurring around midday (Fig. 2a).

#### Patterns of abundance of C. cyanostigma

**Figure 3:**
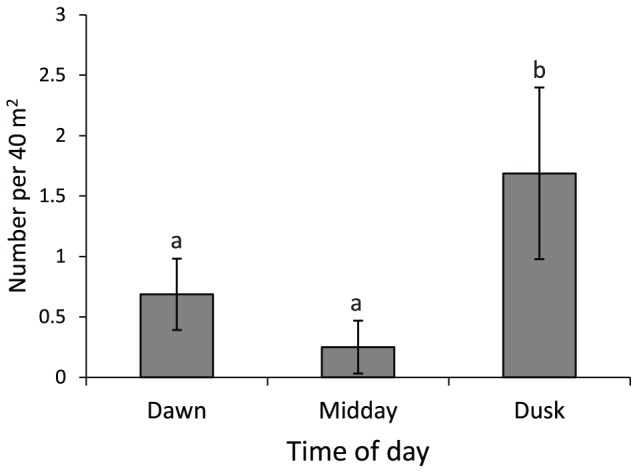
Mean (± SE) number of *Cephalopholis cyanostigma* visible within 20×2 m visual strip transects, at three times of the day: dawn, midday, and dusk. Letters above bars represent Tukey's HSD homogenous subsets. N = 16.

There was a significant difference in the number of *C. cyanostigma* visible at different times of the day (F_2, 45_ = 6.542, p<0.01; Fig. 3). *C. cyanostigma* was significantly more visibly abundant at dusk compared to dawn and midday (Tukey's HSD, p<0.05).

### Behaviour of predators in laboratory

**Figure 4:**
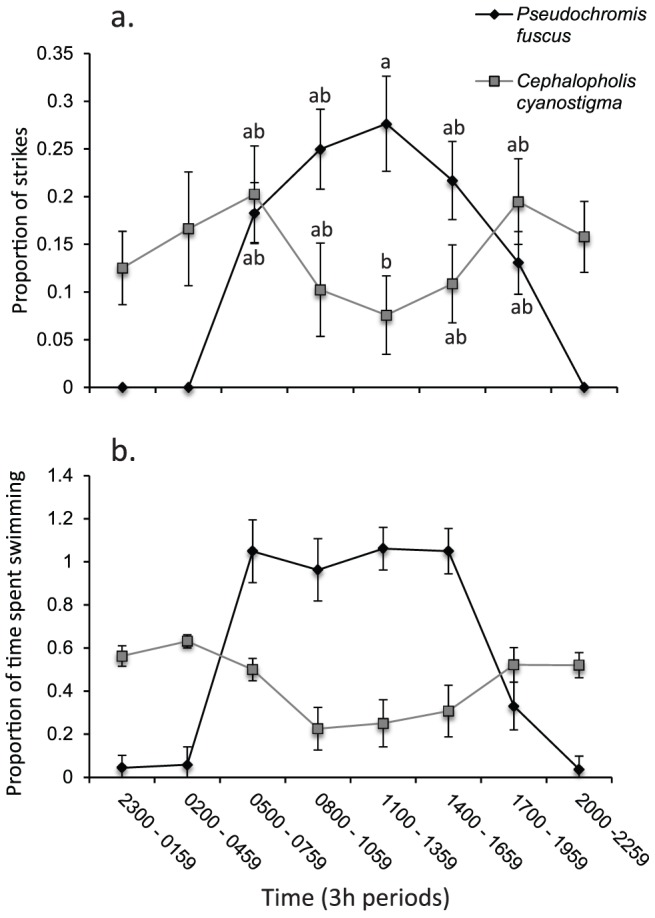
Foraging and activity of *Pseudochromis fuscus* and *Cephalopholis cyanostigma* over a diel cycle within laboratory tanks (see **Fig. 1**). a) Mean (± SE) proportion of daily strikes per 3 h time period, b) mean proportion of time spent swimming versus hiding per 3 h time period. Both variables were arcsin-squart-root transformed for analysis. N = 10 per time per species.

The two species differed in their diel foraging patterns, with a univariate RMANOVA showing a significant interaction between time of day and predator species for the proportion of strikes within daytime hours (F_4, 72_ = 4.473, p<0.01). Tukey's HSD post-hoc comparisons revealed that *P. fuscus* made a significantly (p<0.05) higher proportion of its strikes at midday (11:00–13:59 h) than *C. cyanostigma* (Fig. 4). Examination of the means revealed that *P. fuscus* struck at prey compartments less frequently during the nighttime periods (20:00–04:59 h) than daytime (05:00–19:59 h) and less during the nighttime than *C. cyanostigma* (Fig. 4). Differences between the two species are driven by their opposing diel foraging patterns.

**Figure 5:**
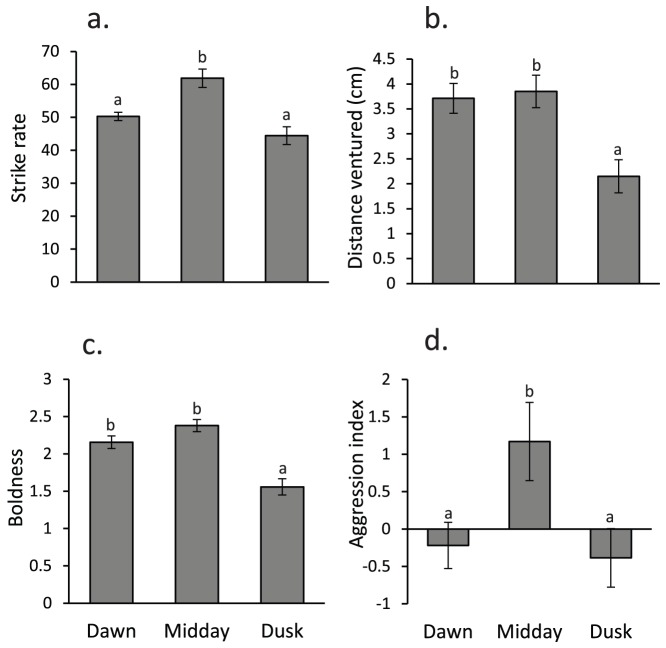
Mean (± SE) behaviour of juvenile *Pomacentrus moluccensis* at three times of the day: dawn, midday and dusk. a) Strike rate per 3 min focal observation, b) relative horizontal distance from coral patch (cm), c) boldness (a value of 0 represents a shy individual, 3 very bold), d) aggression index (the larger the value, the more aggressive). Letters above bars represent Tukey's HSD homogenous subsets of means (p < 0.05 for subsets to differ). N  =  32, 29, 26 (dawn, midday and dusk respectively).

The two predators also differed in their diel activity patterns, with a multivariate RMANOVA revealing a significant interaction between species and time for the proportion of time spent swimming over the entire 24 h day (Pillai's trace_7, 12_ = 0.8957, p<0.0001). Examination of means shows that *P. fuscus* spent a substantially greater proportion of time swimming than *C. cyanostigma* at dawn (05:00–07:59 h), morning (08:00–10:59 h), midday (11:00–13:59 h) and afternoon (14:00–16:59 h) (Fig. 5). In contrast, *C. cyanostigma* was more active than *P. fuscus* during the nighttime periods (20:00–04:59 h) (Fig. 5). Looking at each predator separately, there appears to be no differences in the proportion of time spent swimming between dawn (05:00–07:59 h), morning (08:00–10:59 h), midday (11:00–13:59 h) and afternoon (14:00–16:59 h) for *P. fuscus* (Fig. 5). There does however, appear to be a difference between these time periods and both dusk (17:00–1959 h) and night (20:00–04:59 h) (Fig. 5). In contrast to *P. fuscus*, *C. cyanostigma*, spent a slightly greater proportion of time swimming at night compared to morning and midday (Fig. 4).

Finally, an RMANOVA investigating the effect of replicate start time on the behaviour of predators indicated that there was no significant difference between predator behaviour when videos were started at 10:30 h as opposed to 16:00 h (RMANOVA: proportion of strikes, Pillai's trace_4, 15_ = 0.299, p = 0.225; proportion of time spent swimming, Pillai's trace_4, 15_ = 0.223, p = 0.403). This suggests that hunger level over the course of the 24 diel cycle was not significantly influencing predatory behaviour.

### Behaviour of prey in the field

There was a significant difference in behaviour exhibited by *P. moluccensis* among dawn, midday and dusk (MANOVA, Pillai's Trace_12, 158_ = 0.648, p<0.0001; Fig. 5). Univariate results revealed a significant change with time of day for strike rate (F_2,83_ = 25.941, p<0.0001), aggression (F_2, 83_ = 3.748, p<0.05), boldness (F_2, 83_ = 20.591, p<0.0001) and relative horizontal distances from coral patch (F_2, 83_ = 7.355, p<0.01). Tukey's HSD means comparisons revealed a significant (p<0.05) reduction in strike rate and aggression at dawn and dusk compared to midday. Boldness and relative horizontal distances from coral patch were significantly reduced only at dusk compared to both midday and dawn. The total distance moved and maximum horizontal distance ventured did not differ significantly between the three times of the day (p>0.05), although these variables showed similar patterns to the other response variables.

## Discussion

Few studies have quantified patterns of predator foraging and activity over a diel cycle and related these to activity patterns of their prey in a coral reef ecosystem. Using a combination of field observations and infrared video cameras in the aquaria, we determined the foraging and activity patterns of two common, small-bodied predators of juvenile fish, *C. cyanostigma* and *P. fuscus*, over the full 24 h cycle. Our results show that the two predator species have relatively predictable and yet strikingly different diel activity and foraging patterns. A common prey species, *P. moluccensis*, showed predictable patterns of behaviour that were opposite of those of its key predator, *C. cyanostigma*. The existence of drastically different diel foraging patterns between sympatric predators highlights the complexity of predator-prey interactions on coral reefs, however it seems that even in this complex ecosystem prey fish may respond to key predator activity patterns by displaying opposing behavioural patterns.

### Foraging and activity patterns of resident predators

Small rockcods, such as *C. cyanostigma*, are thought to have a considerable impact on prey populations [51]. Our results suggest that *C. cyanostigma* is a very active hunter with 117 strikes observed in approximately 34 h of observations. This figure is a third-again that obtained by Shpigel and Fishelson (1989) [47] who recorded 50 attacks per 20 h of observation for *Cephalopholis miniata*, and approximately 3.5 times that observed by Martin (1994) [57] who recorded only 20 strikes per 20 h of observation for *Cephalopholis boenak*. The disparity may be a result of species-specific differences and/or variation among geographical areas in which studies were conducted [58]. It is also likely that slight discrepancies in the way a strike was defined could have led to apparent differences between studies. We found that *C. cyanostigma* targets juvenile fish more than invertebrates (63 and 37 strikes total respectively), which is in concordance with other studies conducted on this species [51]. Unfortunately, the strike mode employed by *C. cyanostigma* (rapid engulfing of prey through a combination of ram and suction feeding) made it impossible to confidently determine success of strikes in the field. Our observations can therefore only be used as an estimate of foraging activity and cannot be used to infer actual consumption rates. We suggest this may be a general problem associated with studying predators with similar foraging modes, and that claims of precise consumption rates from behavioural observations should be treated with caution.

The family Serranidae is believed to contain both diurnal and crepuscular species [33], [59], [60]. However, unlike other small serranids which forage at both periods of low light levels, dawn and dusk [47], [57], the present study found that *C. cyanostigma* hunt preferentially at dusk in the field. The number of *C. cyanostigma* visible was also significantly higher at dusk than other times of the day, providing further support for *C. cyanostigma's* increase in activity at this time of day. As *C. cyanostigma* is relatively site attached and rarely moves long distances over a short period of time [61], the lower numbers of visible fish at dawn and midday is likely due to them occupying the inner reef matrix rather than moving to new habitats. Our controlled laboratory observations suggested that *C. cyanostigma* have feeding peaks at both dawn and dusk in the aquaria, and hence it remains possible that *C. cyanostigma* continues to forage within the reef matrix in the field at dawn. A number of studies have suggested that predation by small-bodied coral reef predators is generally most intense at dusk [37], [62]. Further research on the nature of territorial activities in *C. cyanostigma* at different times of the day would be necessary before any strong conclusions to be drawn.

Our controlled aquaria study highlights marked differences in diel patterns of foraging and activity between the two predators. *C. cyanostigma* showed some activity over the whole 24 h cycle with possible crepuscular peaks, while *P. fuscus* was diurnally active, with a foraging peak at midday. The variation in foraging and activity patterns between the two predators is likely to be a product of complex interactions between their visual physiology, trophic position, and potential need for behavioural sleep.

Predators should possess visual systems optimized for the light conditions of the habitat they frequent and the time of day when foraging is most efficient [21]. In support of this theory, diurnal fishes typically have high cone densities throughout their retina, allowing them to maximize motion detection in prey [21]. Little work has been completed on the visual capabilities of either *C. cyanostigma* or *P. fuscus*, however preliminary investigation suggests that *P. fuscus* has a predominantly cone dominated retina (areas of high cell density reaching up to 31,000) (Amira Parker, University of Queensland pers. comm.), suited for daytime vision. While no work has been completed on the retina of *C. cyanostigma*, we would expect this predator to have somewhat intermediate eyes (fewer but larger cones than those found in diurnal eyes and more but smaller cones than are found in nocturnal eyes) to be able to function effectively during changing conditions of dawn and dusk, as found for other crepuscular predators by Munz and McFarland (1973) [21].

Within the framework of these physiological constraints, game-theory models indicate that predator activity patterns will be strongly influenced by the availability and vulnerability of their prey [19], [63]. During crepuscular periods, the reduced visual capabilities of diurnal reef fishes make them relatively easy targets for cryptic ambush predators, providing a good reason for *C. cyanostigma* to feed during crepuscular hours [21], [32]. As *P. fuscus* is also piscivorous, we would expect that crepuscular feeding might convey a similar benefit. However when considering models of predator-prey games we often need to include multiple predator trophic levels [3]. As *P. fuscus* is significantly smaller than *C. cyanostigma* (max size 90 mm TL) [33], *P. fuscus* is likely to be a potential prey for crepuscular predators such as *C. cyanostigma*, and studies have found *P. fuscus* in the guts of *C. cyanostigma* [51] and its congeneric *Cephalopholis boenak* [64]. *P. fuscus*'s midday activity may mean that predation threat has had a major influence on its activity patterns.

The inactivity of *P. fuscus* during the night may be due to its requirement for “behavioural sleep” [14]. This occurs when a fish remains quiet and in a typical rest posture for long periods of the 24 h day, and is thought to be important for refreshing memory circuits in the brain [14]. Given the complex decision making abilities demonstrated by *P. fuscus* [65], sleep would be highly advantageous for preserving knowledge of the relationship between perceived events and their consequences [14]. Various authors have also pointed to the link between behavioural sleep and the presence of fixed circadian rhythms, which may act as an evolutionary constraint inhibiting plasticity in diel activity patterns [11], [14]. Whether circadian rhythmicity may in fact restrict *P. fuscus* to display activity only in daytime hours requires further investigation. Behavioural sleep does not appear to be required by *C. cyanostigma*, with its continual swimming characteristic of species not specialised for foraging at a particular time of the day (for a review, see [66]). Few non-sleeping fishes show strong and fixed circadian rhythms [14], suggesting that *C. cyanostigma* may be more capable than *P. fuscus* of responding to potential changes in its prey's diel activity patterns.

### Activity and foraging patterns of prey

Our field study revealed that *P. moluccensis* forages most during the middle of the day, with reductions at both dawn and dusk. This is similar to the diurnal pattern of foraging described for other damselfish species [37], [67], [68]. This diel pattern of behaviour opposes the dusk-active pattern documented for *C. cyanostigma*, and found for a number of other small-bodied reef predators [37], [57]. There is therefore reason to believe that *P. moluccensis's* diel behavioural pattern is strongly influenced by predation risk, as has been suggested anecdotally for a number of prey fish species [26], [69]. Yet food availability and capture efficiency is also likely to be highly important in influencing the diel periodicity of *P. moluccensis*. The diel behaviour of *P. moluccensis* is likely due to complex interaction between maximising food intake and minimising predation risk, similar to other teleosts [9], [70].

Foraging theory suggests that the increase in feeding by *P. moluccensis* at midday would be to exploit higher prey densities, maximize prey capture rates, and minimize prey-search time [16], [71]. Although zooplankton abundance on coral reefs is generally highest during crepuscular hours [68], [72], zooplanktivorous damselfishes typically have retinal structures that function optimally at high light levels [21], [73]. This visual mode tends to maximise resolution and motion detection in the daytime, but comes at the expense of reduced night vision and means fish have to spend longer searching for prey at dimmer light levels [10]. Yet the decrease in light intensity at dusk also increases the risk of predation for damselfishes because the ability to detect predators is reduced [74].

Therefore, the reduction in foraging by *P. moluccensis* at dawn and dusk may also be a response to predation threat. Foraging can put fish at considerable risk of predation as handling food can impair the visual field around a fish for some seconds [75]. Reduction in feeding rate is a common response to predation threat in coral reef prey fish [76]. Investigation of predator foraging patterns, however, suggest that *P. moluccensis* opposes foraging patterns of *C. cyanostigma* at dusk and not at dawn. Whether reduced foraging at dawn may also be due to increased predation pressure from other crepuscular mesopredators at this time requires further investigation. Furthermore, the possibility also remains that *C. cyanostigma* is foraging within the reef matrix at dusk and continues to influence *P. moluccensis* during this time. A study by Rickel and Genin (2005) [38] shows support for the importance of predation risk in influencing the behaviour of prey fish. When placed in a largely predator free environment, the humbug damselfish, *Dascyllus marginatus* fed, albeit at reduced levels, under light intensities much lower than the level at which they emerge from and retreat to shelter in the field with predators present [38]. The reduction in foraging in the field at an earlier time than that observed in predator controlled environments provides strong evidence that behaviour by diurnal prey fish may also be a response to predation threat by piscivorous fishes.

In contrast to *C. cyanostigma*, *P. moluccensis* did not oppose the activity or foraging patterns of *P. fuscus*. The most parsimonious explanation is that given the gape limit and size preference of *P. fuscus*, our focal *P. moluccensis* (at ∼25.02 mm SL) were too large for *P. fuscus* to consume [49]. Predation from *P. fuscus* would therefore pose a much lower (or no) threat to juvenile *P. moluccensis* compared to the risk posed by *C. cyanostigma*. Whether activity patterns similar to those exhibited by 1-month post-settlement *P. moluccensis* are seen in smaller recruit fishes (within 14 days of settlement) which are subject to risk from *P. fuscus* [49], will be an interesting area for further investigation.

Prey have the capacity to rapidly learn diel patterns of predation risk and respond to such risk accordingly [9], [77]. In environments where the habitat is topographically complex and composition of predators is spatially and temporally variable, prey benefit from having a flexible mechanism of predator learning, reinforcing and forgetting [78]–[81]. The opposing pattern of prey behaviour relative to that of a key resident predator in the present study suggests that predation risk may be predictable enough in the short term for prey to learn the temporal patterns of predation risk [9], [82], [83]. A recent laboratory study has shown that *P. moluccensis* learns a predictable temporal pattern of risk (in this instance the predictable occurrence of chemical alarm cues) after 6 days of exposure and modifies its activity to minimize the risk of predation [84]. Other studies have also emphasized that this species has a highly sophisticated learning mechanism that is capable of recognizing multiple unknown predators upon recruitment to a reef from a single learning event [79]. These studies suggest that risk avoidance may be largely responsible for the marked reduction in activity around dusk found in the field observations.

## Conclusions

The current study indicates that temporal predation risk may be relatively predictable in coral reef habitats, and dependent on the locally abundant species of resident predator. Small-bodied, highly territorial predators, such as *P. fuscus* and *C. cyanostigma* are patchily distributed among habitat types and thus prey may be exposed to different daily patterns of risk depending on which predators are in their habitat patches [44]. The current findings suggest that the activity patterns of damselfishes such as *P. moluccensis* may be in response to increased predation risk at particular times of the day, as has been indicated in a number of largely anecdotal studies [21], [25], [67]. Evidence suggests that these small prey have highly sophisticated ways of learning the identity of relevant predators and adjust this information for current relevance as predators change with the growth of prey [85] and/or through imposed or chosen habitat shifts [86]. This honed perception of predation risk is a likely a major determinant of changes in the diel pattern of activity of prey (and to a lesser extent mesopredators) and how these patterns change with ontogeny.

## Supporting Information

File S1 Contains the following files: Table S1. Data for Figure 2. Table S2. Data for Figure 3. Table S3. Data for Figure 4. Table S4. Data for Figure 5.
(DOCX)
