# Supplementary material for: Temporal Links in Daily Activity Patterns between Coral Reef Predators and Their Prey
Source: PLoS One. 2014 Oct 29;9(10):e111723. doi: 10.1371/journal.pone.0111723 (PMC4213059; doi:10.1371/journal.pone.0111723)
Supplement: File S1 — Contains the following files: Table S1. Data for Figure 2. Table S2. Data for Figure 3. Table S3. Data for Figure 4. Table S4. Data for Figure 5. (DOCX) [file pone.0111723.s001.docx]

Supplementary data file – Bosiger & McCormick - Temporal links in daily activity patterns between coral reef predators and their prey

Table S1. Data for Figure 2. Diel variation in behaviour of *Cephalopholis cyanostigma* and *Pseudochromis fuscus* (means ± SE) for three times of the day: dawn, midday, and dusk. 2a) Total strikes per minute at fish for *C. cyanostigma* and *P. fuscus*, 2b) proportion of time spent swimming (as opposed to hiding and stationary) by *C. cyanostigma*, 2c) total distance (m) moved per minute by *C. cyanostigma*, and 2d) aggression index (+ aggression, - avoidance) for *C. cyanostigma*. N = 26, 21, 23 for dawn, midday and dusk respectively for *C. cyanostigma*, while N = 6, 6, and 8 for *P. fuscus*. Note that the definition of the time intervals for the two species differ slightly (see text for details). Data for *P. fuscus* in Fig. 2a from Feeney et al. (2012).

| Figure | Species | Time of day | Dawn | Midday | Dusk |
| --- | --- | --- | --- | --- | --- |
| 2a | *C. cyanostigma* | Strikes on fish | 0.028 | 0.012 | 0.053 |
|  | *C. cyanostigma* | SE | 0.007 | 0.008 | 0.007 |
| 2a | *P. fuscus* | Strikes on fish | 0.146 | 0.254 | 0.138 |
|  | *P. fuscus* | SE | 0.033 | 0.030 | 0.029 |
| 2b | *C. cyanostigma* | Time spent swimming | 0.366 | 0.123 | 0.337 |
|  | *C. cyanostigma* | SE | 0.034 | 0.038 | 0.036 |
| 2c | *C. cyanostigma* | Total distance moved /min | 1.593 | 0.569 | 1.279 |
|  | *C. cyanostigma* | SE | 0.143 | 0.159 | 0.152 |
| 2d | *C. cyanostigma* | Aggression index | 0.383 | 0.106 | 0.567 |
|  | *C. cyanostigma* | SE | 0.086 | 0.098 | 0.091 |

Table S2. Data for Figure 3. Mean (± SE) number of *Cephalopholis cyanostigma* visible within 20 x 2 m visual strip transects, at three times of the day: dawn, midday, and dusk. N = 16.

| Time of day | Mean | SE |
| --- | --- | --- |
| Dawn | 0.688 | 0.151 |
| Midday | 0.250 | 0.112 |
| Dusk | 1.688 | 0.362 |

Table S3. Untransformed data for Figure 4. Foraging and activity of *Pseudochromis fuscus* and *Cephalopholis cyanostigma* over a diel cycle within laboratory tanks (see Fig. 1). 4a) Mean (± SE) proportion of daily strikes per 3 h time period, 4b) mean proportion of time spent swimming versus hiding per 3 h time period. N = 10 per time per species.

| Figure | Species | Variable | Time of day | Average | SE |
| --- | --- | --- | --- | --- | --- |
| 4a | *P. fuscus* | Proportion of strikes | 0500 - 0759 | 0.042 | 0.011 |
| 4a | *P. fuscus* |  | 0800 - 1059 | 0.075 | 0.017 |
| 4a | *P. fuscus* |  | 1100 - 1359 | 0.094 | 0.024 |
| 4a | *P. fuscus* |  | 1400 - 1659 | 0.060 | 0.015 |
| 4a | *P. fuscus* |  | 1700 - 1959 | 0.026 | 0.008 |
| 4a | *P. fuscus* |  | 2000 -2259 | 0 | 0 |
| 4a | *P. fuscus* |  | 2300 - 0159 | 0 | 0 |
| 4a | *P. fuscus* |  | 0200 - 0459 | 0 | 0 |
| 4a | *C. cyanostigma* |  | 0500 - 0759 | 0.062 | 0.019 |
| 4a | *C. cyanostigma* |  | 0800 - 1059 | 0.031 | 0.018 |
| 4a | *C. cyanostigma* |  | 1100 - 1359 | 0.025 | 0.013 |
| 4a | *C. cyanostigma* |  | 1400 - 1659 | 0.033 | 0.013 |
| 4a | *C. cyanostigma* |  | 1700 - 1959 | 0.054 | 0.014 |
| 4a | *C. cyanostigma* |  | 2000 -2259 | 0.036 | 0.012 |
| 4a | *C. cyanostigma* |  | 2300 - 0159 | 0.028 | 0.010 |
| 4a | *C. cyanostigma* |  | 0200 - 0459 | 0.056 | 0.026 |
| 4b | *P. fuscus* | Proportion of time spent swimming | 0500 - 0759 | 0.737 | 0.066 |
| 4b | *P. fuscus* |  | 0800 - 1059 | 0.661 | 0.067 |
| 4b | *P. fuscus* |  | 1100 - 1359 | 0.752 | 0.044 |
| 4b | *P. fuscus* |  | 1400 - 1659 | 0.743 | 0.049 |
| 4b | *P. fuscus* |  | 1700 - 1959 | 0.128 | 0.032 |
| 4b | *P. fuscus* |  | 2000 -2259 | 0.010 | 0.009 |
| 4b | *P. fuscus* |  | 2300 - 0159 | 0.009 | 0.006 |
| 4b | *P. fuscus* |  | 0200 - 0459 | 0.019 | 0.015 |
| 4b | *C. cyanostigma* |  | 0500 - 0759 | 0.241 | 0.046 |
| 4b | *C. cyanostigma* |  | 0800 - 1059 | 0.109 | 0.068 |
| 4b | *C. cyanostigma* |  | 1100 - 1359 | 0.176 | 0.075 |
| 4b | *C. cyanostigma* |  | 1400 - 1659 | 0.217 | 0.089 |
| 4b | *C. cyanostigma* |  | 1700 - 1959 | 0.27594444 | 0.0639656 |
| 4b | *C. cyanostigma* |  | 2000 -2259 | 0.26055556 | 0.0500758 |
| 4b | *C. cyanostigma* |  | 2300 - 0159 | 0.29105556 | 0.04406127 |
| 4b | *C. cyanostigma* |  | 0200 - 0459 | 0.35083333 | 0.02857261 |

Table S4. Data for Figure 5. Mean (± SE) behaviour of juvenile *Pomacentrus moluccensis* at three times of the day: dawn, midday and dusk. 5a) Strike rate per 3 min focal observation, 5b) aggressive index (the larger the valuse, the more aggressive), 5c) boldness (a value of 0 represents a shy individual, 3 very bold), 5d) relative horizontal distance from coral patch (cm) (a value of 0 represents 100% of time spent within shelter), 5e) maximum horizontal distance ventured in a 3 min observation, and f) total distance moved in a 3 min observation. N = 32, 29, 26 (dawn, midday and dusk respectively).

| Figure | Variable | Dawn | Midday | Dusk |
| --- | --- | --- | --- | --- |
| 5a | Strikes/min | 50.281 | 61.897 | 44.462 |
|  | SE | 1.232 | 2.784 | 2.668 |
| 5b | Distance ventured | 3.712 | 3.851 | 2.150 |
|  | SE | 0.300 | 0.325 | 0.330 |
| 5c | Boldness | 2.156 | 2.379 | 1.558 |
|  | SE | 0.085 | 0.081 | 0.106 |
| 5d | Aggressive index | -0.219 | 1.172 | -0.385 |
|  |  | 0.310 | 0.523 | 0.393 |
